# T follicular helper and B cell crosstalk in tertiary lymphoid structures and cancer immunotherapy

**DOI:** 10.1038/s41467-022-29753-z

**Published:** 2022-04-26

**Authors:** Soizic Garaud, Marie-Caroline Dieu-Nosjean, Karen Willard-Gallo

**Affiliations:** 1 Molecular Immunology Laboratory, Institut Jules Bordet, Université Libre de Bruxelles, Brussels, Belgium; 2 Sorbonne University UMRS1135, Inserm U1135, Laboratory “Immune microenvironment and immunotherapy”, Centre d’Immunologie et des Maladies Infectieuses Paris (CIMI-Paris), Paris, France

**Keywords:** Tumour immunology, Cancer immunotherapy

## Abstract

Tumor-infiltrating lymphocytes (TILs) are critical in the elimination of cancer cells, a concept highlighted by recent advances in cancer immunotherapy. Significant evidence reveals that their organization in tertiary lymphoid structures together with specific subpopulation composition/balances stimulates cellular crosstalk and anti-tumor immunity in patients.

Across most solid tumor types, data support an important link between higher densities of TILs and better clinical outcomes. TIL composition however varies dynamically, with the extent and balance of immune cell subpopulations changing both within and between tumors. There is robust data showing that ectopic lymphoid tissues in the form of tertiary lymphoid structures (TLS; Fig. 1A) are present in the tumor microenvironment (TME) of most cancer types, although not in all patients^1^. Mature TLS provide strategic sites for local immune cell interactions with an architecture resembling that of secondary lymphoid organs, including a distinct T cell zone adjacent to a B cell follicle. Based on their cellular composition and degree of organization, different TLS maturation stages have been reported in human tumors^2^. Current thinking is that TLS are generated from dense lymphocytic aggregates, largely composed of T and B cells, commonly observed in TIL-positive tumors of most cancer types. Early TLS lack a dendritic cell (DC) scaffold and vascularization. Immature TLS (primary lymphoid follicles) are largely composed of a T cell zone and B cell follicle with DC but no germinal center (GC). Mature TLS (secondary lymphoid follicles) incorporate lymphatic vessels, a segregated T cell zone including T follicular helper (Tfh) cells, mature DC, and a B cell follicle that includes mantle and GC B cells (active GC contains proliferating B cells), follicular DC and macrophages^2–5^. Some TLS are thought to be defective or attenuated and while they have a defined T cell zone, B cell follicle, and lymphatic vessels, they characteristically lack an active GC and contain higher numbers of regulatory cells^6^, including T regulatory (Treg), T follicular regulatory (Tfr; Treg specific for Tfh) and B regulatory (Breg) cells.

**Fig. 1:**
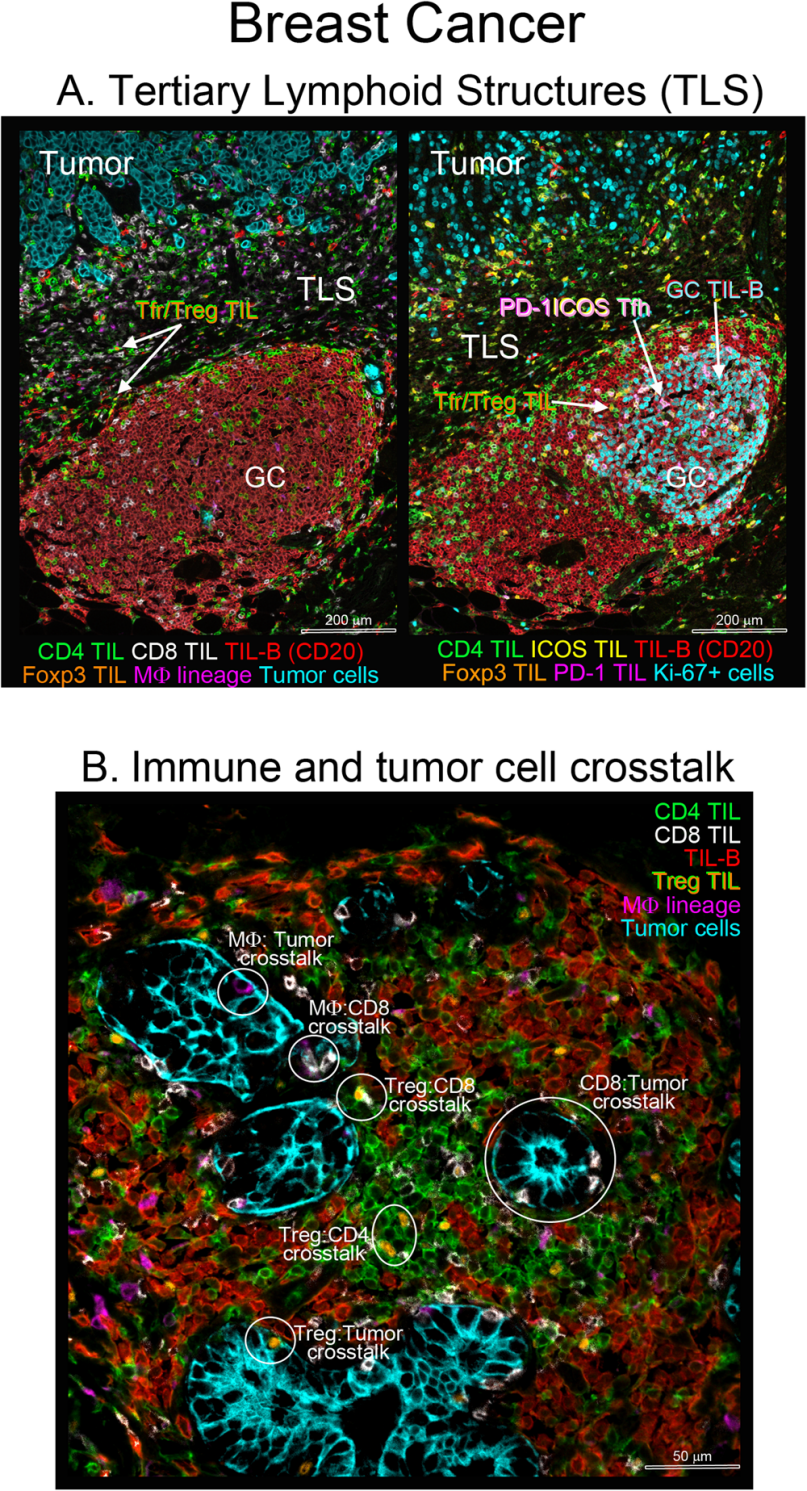
Tertiary lymphoid structures and immune cell crosstalk in the breast cancer immune microenvironment. Breast tumor tissue sections from surgical resections (formalin-fixed and paraffin-embedded; FFPE) analyzed using multiplex immunohistochemistry. **A** Upper left image: region of an untreated primary breast tumor showing multiplex IHC-stained tumor cells (pan CK, cyan) with immune cells in stromal localized TLS. TIL include CD4 (green) and CD8 (white) T cells, B cells (CD20, red), Tfr/Treg [Foxp3 (orange nucleus) plus CD4 (green membrane)] and macrophage lineage (CD68, magenta); upper right image: consecutive section of the same tumor region showing CD4 T cells (green), B cells (CD20, red), ICOS+ cells (yellow), Tfh [their pink/white color is a combination of three surface markers: PD-1 (magenta), ICOS (yellow) and CD4 (green)], Tfr/Treg [Foxp3 (orange nucleus) plus CD4 (green membrane)] and proliferating cells (Ki-67+, cyan; both B cells and tumor cells). **B** Enlarged region of TIL surrounding tumor islets in a residual tumor surgically resected following pre-surgical treatment. Multiplex IHC-stained tumor cells (pan CK, cyan) and TIL including CD4 (green), CD8 (white), B cells (CD20, red), Treg [Foxp3 (orange nucleus) plus CD4 (green membrane)] and macrophage lineage (CD68, magenta) are shown with examples of the crosstalk between these cells shown in circles (white). mIHC slides were scanned at ×20 magnification.

The communication and cooperation between cells in the structured immune microenvironment of an active TLS fosters naïve or memory cell (re-)activation leading to effector cell expansion, something not readily achieved by random TIL migrating through the tumor bed. Accumulation of regulatory cells as immune responses progress can also dampen immune activities and potentially lead to TLS inactivation or dormancy^6^. A TLS presence in the TME is generally thought to reflect the capacity of a patient’s immune response to recognize and respond immunologically to their tumor as an anomaly. The prognostic value of having more than two mature TLS, containing active T cell zones plus B cell follicles with a GC, is significantly better than inactive or single-zone TLS^4,6^. Thus, just as conventional immune responses in secondary lymphoid organs protect us on a daily basis, the interplay and coordinated activity of TLS-associated immune functions in the TME generate anti-tumor immunity and improve cancer patient survival.

Tumor-infiltrating B cells (TIL-B) are detected in only a minority of patients within a given tumor type, but when amply present they are generally associated with better clinical outcomes and TLS. Principally enriched at the invasive margin, TIL-B tend to form dense aggregates (pre-TLS B cell follicles?) or reside in TLS, both typically facing the tumor bed. In contrast to tumor-infiltrating T cells, which are primarily antigen-experienced cells, all B cell differentiation stages are detectable in the TME^5^. In secondary lymphoid organs, the GC functions to produce affinity-matured and class-switched B cells that recognize cognate antigen and thereby generate memory B cells and durable humoral immunity. In the B cell follicles of cancer-associated TLS, local antigen-driven responses and antibody affinity maturation are observed together with B cell clonal amplification, somatic hypermutation, and class-switch recombination^7^. GC B cells have been tightly correlated with global TIL that are characterized by Tfh TIL, Th1- oriented CD4 TIL, antibody-secreting TIL-B, and long-term survival, reflecting a mature TLS presence^5,6^.

Closely linked to TIL-B functionality are their primary helpers, Tfh TIL, a hallmark of TLS and anti-tumor immune responses in a variety of tumor types^8^. Tfh:B partnering was first recognized for its role in providing help in the GC for memory and antibody-producing B cell differentiation. In addition to Tfh delivering T-dependent B cell help, specialized CXCL13-producing Tfh cells have been shown to guide CXCR5^+^ lymphocyte migration under both normal and pathological conditions and promote TLS and GC formation at chronic inflammatory sites^3^. Tfh TIL are easily recognized in the GC by their high PD-1 surface expression^6,9^. The level of PD-1 expression on Tfh cells is known to regulate their expansion, location, and function. Moreover, active TLS are distinguished by functional PD-1^hi^ Th1-oriented Tfh TIL expressing IFNγ, IL-21, and CXCL13^6^. High PD-1 expression is also detected on Th1-oriented CD8 TIL producing IFNγ and granzyme B. These data suggest that high surface PD-1 on TLS-resident Tfh TIL might have less to do with exhaustion and more to do with modulating their activities and functionality. Alternatively, PD-1 may not engage in these situations either due to PD-L1 occupancy by CD80 or the lack of PD-L1 expression on local antigen-presenting DC or B cells. Under these conditions, CD80 could play a critical role in the TLS by limiting PD-1/PD-L1 engagement, with the CD80/PD-L1 axis favoring T cell activation through CD28.

The role of this Tfh:B tango in tumor immunity has been undervalued until recently. Mounting evidence over the past decade suggests that the major TIL subpopulations (CD4, CD8, and CD19/20) encompass varying subset balances with suppressive (pro-tumor) or effector (anti-tumor) phenotypes whose functions are influenced by the surrounding TME^10^. Natural or treatment-induced immune activation or suppression may dictate the prevailing balance between pro- or anti-tumor immune cell crosstalk within a given tumor (Fig. 1B) thereby guiding specific immune responses and encounters. Key anti-tumor effector activities include antibody-dependent cell cytotoxicity, complement activation, antibody-mediated tumor cell phagocytosis, antigen presentation, T cell activation, cytokine secretion, and direct tumor killing by TIL, including CD8, NK, B cells, and/or macrophages^11^. Once TIL mature in the protected arena of a TLS, they are expected to migrate into the tumor bed where they should, at least initially, exert effector functions. This could be followed by a rise, both in the TLS and tumor bed, in immunosuppressive regulatory subpopulations that function to dampen responsiveness but paradoxically contribute to tumor growth. Other antigen-experienced TIL may directly migrate to the periphery as long-lived memory cells where their role is to recognize disseminated tumor cells and kill or exert pressures that maintain their quiescence.

PD-1/PD-L1 interactions between immune and tumor cells are known to specifically interfere with anti-tumor immunity and the therapeutic blocking of these immune checkpoints underlies the remarkable benefit observed in a minority of cancer patients receiving immunotherapy. Some patients have tumor cells that do not express PD-L1 yet they still respond to treatment with anti-PD-1/PD-L1 inhibitors^12^, suggesting that immune:immune cell interactions may be a key responsive target of anti-PD-1/PD-L1 therapies^12^. Contact in tumors between PD-1^hi^ Tfh and PD-1^int^ Tfr TIL in the GC and/or PD-1^int^ Treg TIL elsewhere with PD-L1^+^ Breg, PD-L1^+^ DC or PD-L1^+^ macrophages potentially are the object of PD-1/PD-L1 antibody blocking drugs. Releasing the PD-L1 blockade on PD-1^+^ effector TIL and restoring critical interactions, such as Tfh:B and Tfh:CD8 pairs, in the tight confines of a TLS, could reactivate waning immune responses and/or restore TLS from dormancy/inactivation. Supporting evidence that blocking the PD-1/PD-L1 pathway can revive T cell function comes from virology, once again demonstrating important parallels between cancer and chronic viral infection^13^. This revitalization could underlie the impressive responses of some cancer patients treated with checkpoint inhibitors targeting the PD-1/PD-L1 pathway.

Two Phase II trials in 2018 (lung cancer patients treated with Nivolumab and melanoma patients treated with Nivolumab or Nivolumab+Ipilimumab), found that responder versus non-responder patients had higher TLS densities^14,15^. In addition, higher expression of T and B cell markers, including those associated with active immune responses, were detected in responders after treatment^15^. TIL and TLS densities were also shown as predictive of chemotherapy responses in breast cancer, particularly in the high-grade HER2-positive and triple-negative subtypes^16,17^. Finally, a positive association between TLS and TIL-B for disease-free and overall survival in patients treated with immunotherapy was impressively demonstrated in melanoma, metastatic kidney cancer, and sarcoma^18–20^. TLS arise at sites of chronic inflammation due to a persistent imbalance between the recruitment and clearance of immune cells. Higher TIL recruitment and TLS formation in tumors may promote local cellular interactions that can generate antigen-specific immune responses. Unfortunately, an association between immunotherapy responsiveness, TIL, and TLS has not been observed in some patients, such as melanoma patients with a BRAF mutation. This suggests that heterogeneity in the immune microenvironment, reflecting a patient’s distinct mutational and neoantigen profiles or critical balances between active and inactive TLS in their TME, may be principal drivers of responsiveness to immunotherapy. Interactions between T and B cell subpopulations with one another and other immune cells are likely fostered in the TLS microenvironment similar to their daily operations in lymph nodes. These activities would be expected to stimulate functional and protective cooperation; however, if tumor-specific antigens are not present then any inflammatory responses generated would not be primed to eradicate tumor cells with specificity.

Open questions remain, including the identity of TLS-resident immune cell subpopulations that are the primary responsive target(s) of PD-1/PD-L1 immunotherapy. Are they functional PD-1^hi^ Tfh, functional PD-1^int^ Tfr/Treg, PD-L1^+^ or regulatory TIL-B, PD-L1^+^ DC or PD-L1^+^ macrophages? Or is there reactivation of non-functional PD-1^hi^ Tfh or PD-1^hi^ CD8 TIL? When using drugs to target the checkpoint molecule CTLA-4, highly expressed on regulatory T cells, is this pathway driving Treg and Tfr to release their hold on Th and Tfh TIL, respectively? Does targeting these important interactions change the balance of pro- and anti-tumor effectors sufficiently to promote interactions linked with active adaptive immune responses and the generation of immunological memory? Ongoing research efforts not only in cancer but also in chronic viral infections, autoimmune diseases, and other related pathologies will undoubtedly answer many of these questions while simultaneously increasing our understanding of the delicate balance needed to generate effective and durable TLS-facilitated anti-tumor immunity in patients.
